# Effect of Laterally Wedged Insoles on the External Knee Adduction Moment across Different Reference Frames

**DOI:** 10.1371/journal.pone.0138554

**Published:** 2015-09-23

**Authors:** Satoshi Yamaguchi, Masako Kitamura, Tomohiro Ushikubo, Atsushi Murata, Ryuichiro Akagi, Takahisa Sasho

**Affiliations:** 1 Department of Orthopaedic Surgery, Graduate School of Medicine, Chiba University, Chuo-ku, Chiba, Japan; 2 Division of Rehabilitation Medicine, Chiba University Hospital, Chuo-ku, Chiba, Japan; 3 Division of System Development, Anima Corp, Chofu, Tokyo, Japan; University of Pennsylvania, UNITED STATES

## Abstract

**Objective:**

Biomechanical effects of laterally wedged insoles are assessed by reduction in the knee adduction moment. However, the degree of reduction may vary depending on the reference frame with which it is calculated. The purpose of this study was to clarify the effect of reference frame on the reduction in the knee adduction moment by laterally wedged insoles.

**Methods:**

Twenty-nine healthy participants performed gait trials with a laterally wedged insole and with a flat insole as a control. The knee adduction moment, including the first and second peaks and the angular impulse, were calculated using four different reference frames: the femoral frame, tibial frame, laboratory frame and the Joint Coordinate System.

**Results:**

There were significant effects of reference frame on the knee adduction moment first and second peaks (*P* < 0.001 for both variables), while the effect was not significant for the angular impulse (*P* = 0.84). No significant interaction between the gait condition and reference frame was found in either of the knee adduction moment variables (*P* = 0.99 for all variables), indicating that the effects of laterally wedged insole on the knee adduction moments were similar across the four reference frames. On the other hand, the average percent changes ranged from 9% to 16% for the first peak, from 16% to 18% for the second peak and from 17% to 21% for the angular impulse when using the different reference frames.

**Conclusion:**

The effects of laterally wedged insole on the reduction in the knee adduction moment were similar across the reference frames. On the other hand, Researchers need to recognize that when the percent change was used as the parameter of the efficacy of laterally wedged insole, the choice of reference frame may influence the interpretation of how laterally wedged insoles affect the knee adduction moment.

## Introduction

Knee osteoarthritis is one of the most common orthopedic diseases, affecting up to 30% of individuals over the age of 65 [1]. Biomechanical forces are associated with the pathogenesis of knee osteoarthritis [2, 3]. The external knee adduction moment (KAM) is a valid and reliable indicator of medial compartment load [4, 5], and correlates to knee pain [6] and radiographic disease severity [7]. It is also a predictor of initiation and progression of the disease [8]. Thus, it offers a potential target for treatment strategies to reduce pain and slow disease progression.

Laterally wedged insoles were first proposed by Tomatsuri et al. to treat medial knee osteoarthritis [9]. Studies have shown that they improve pain and function, and their use is recommended by 13 out of 14 guidelines [10] as a low-cost and safe intervention with high patient compliance [11]. Biomechanical studies report that lateral wedges reduce the KAM during gait in healthy participants and patients with medial knee osteoarthritis. However, the average amount of reduction in the KAM varied among studies, ranging from 5.6% to 12.6% in healthy participants and 2.1% to 11.9% in patients with knee osteoarthritis [12]. Additionally, biomechanical responses to laterally wedged insoles were not consistent across individuals. For example, the KAM increased while using the insoles in 13% to 18% of knee osteoarthritis patients, while it decreased in other patients [13, 14].

Despite widespread use of the KAM as an indicator of medial knee joint load, there is no standard anatomical reference frame about which to express the KAM. Moments can be expressed using four possible reference frames: the distal segment coordinate system, the proximal segment coordinate system, the laboratory coordinate system, which is along the plane of progression, and the Joint Coordinate System [15–17]. Other possible reference frames have been reported, such as both proximal and distal reference frames [18], and the Dual-Euler basis [19]. Several studies have shown that expressed KAM values are different depending on the reference frame [15, 16, 18, 20]. However, the effect of the reference frame on the reduction of the KAM in response to laterally wedged insoles has not been studied. Indeed, many studies investigating the biomechanical effect of laterally wedged insoles did not clearly address which reference frame was used, which makes it difficult to compare the KAM values and the effect of different insoles between studies.

The purpose of this study was to clarify the effect of reference frame on the reduction of the KAM while using laterally wedged insoles during gait. We hypothesized that reduction of the KAM would be dependent on the reference frame.

## Methods

### Participants

Twenty-nine healthy participants (9 females and 20 males) with a mean ± standard deviation (range) age of 28 years ± 4 (21–37) years participated in this study. Participants with neurological disorders, systemic inflammatory disease, a history of disorder or trauma in the lower extremity, or osteoarthritis were excluded. We excluded participants with neutral or valgus knee alignment [21] because laterally wedged insoles are generally given to knee OA patients with varus knees. Knee alignment was visually assessed using a validated instrument tool. Briefly, knee alignment was classified into 5 grades according to the standard drawing. Knees with “neutral”, “mild valgus”, and “severe valgus” were excluded. The mean height, weight and body mass index were 166 cm ± 9 (141–182) cm, 59 kg ± 10 (38–81) kg and 21 ± 2 (17–27), respectively.

### Ethics statement

This study was approved by the Research ethics committee of the graduate school of medicine, Chiba University, with written informed consent obtained from all participants.

### Marker placement, anatomical coordinate system and gait measurement

Reflective markers of 5-mm diameter were attached to the right lower leg using double-sided tape, and anatomical coordinate systems in the femur, tibia and rear foot were defined. For the femoral segment, the superoinferior axis was defined by the line connecting the hip and knee joint centers. The hip joint center was defined using the anterior superior iliac spine and the posterior superior iliac spine [22]. The knee joint center was the midpoint between the medial femoral condyle and the lateral femoral condyle. The anteroposterior axis was perpendicular to the plane defined by the superoinferior axis and the transcondylar axis. The mediolateral axis was mutually perpendicular. For the tibial segment, the superoinferior axis was defined by the line connecting the ankle and knee centers. The ankle joint center was defined as the midpoint between the medial malleolus and the lateral malleolus. The anteroposterior axis was perpendicular to the plane defined by the superoinferior axis and the transmalleolar axis. The mediolateral axis was mutually perpendicular [23]. For the rear foot segment, a midsagittal plane was defined by the posterior distal aspect of the heel and the posterior proximal aspect of heel, and the midpoint between the sustentaculum tali and the lateral calcaneus. The anteroposterior axis was defined as the line in the midsagittal plane and parallel to the floor in the standing posture. The superoinferior axis was perpendicular to the anteroposterior axis in the midsagittal plane. The mediolateral axis was mutually perpendicular [24].

A three-dimensional motion analysis system (Locus 3D MA-3000, Anima Corp, Tokyo, Japan) was used with 12 infrared cameras to capture and analyze the motion of the pelvis, femur, tibia and the rearfoot segments with a sampling frequency of 100 Hz. Two force plates (MG-100, Anima, Tokyo, Japan), in synchrony with the cameras, were used to capture ground reaction forces and identify heel-contact and toe-off of the stance phase with a sampling frequency of 100 Hz. Raw coordinate data from the force plates were smoothed using a Butterworth filter [25] with a cutoff frequency of 20 Hz [26]. Static calibration was performed with the participant standing at a relaxed position to define the femur, tibia and foot coordinate systems, and to identify the hip, knee and ankle centers. Participants were asked to walk at a comfortable speed along an 8-m walkway. After three accommodation gaits, five successful gait trials for each gait condition were collected. A successful trial required the participant’s foot to land on the center of the force plate without any interference with their gait, and the walking speed to vary less than 5% from that of the first trial.

### Insole

Participants performed gait trials in three conditions: barefoot, a laterally wedged insole of 6° inclination along full length of the foot [27] and a flat insole of 5-mm thickness as a control. The length of the insole was adjusted to fit each participant. The insoles were directly attached to the participants’ soles bilaterally with double-sided tape, and the participants walked on the walkway with the insoles, without wearing shoes [27]. The barefoot gait was performed first, and the two insole conditions were tested in a randomized order.

### Calculation of external knee adduction moment

The KAM was calculated using a standard inverse dynamics approach, incorporating the three-dimensional location of each segment, inertial parameters of the limb segment and the ground reaction forces using built-in software of the motion analysis system [28]. Moments were normalized to body weight and height, and expressed as a function of % stance of gait. Data of the five trials from each participant were averaged to make a participant mean.

The KAMs were expressed in four different reference frames: femoral frame (FF), distal tibial frame (TF), laboratory frame (LF) and the Joint Coordinate System (JCS). We chose the four reference frames according to Brandon et al. [15]. Although the proximal tibial frame can also be used to calculate KAM [18], it has been shown that moments calculated using the proximal tibial frame are similar to those calculated using the JCS [18]. Thus we used only the distal tibial frame to provide the largest possible differences between reference frames. The variables of interest were the peak adduction moment in the first and second half of stance (first peak and second peak) and knee adduction angular impulse during stance [4–8].

### Statistics

Effects of the laterally wedged insole and reference frame, as well as their interaction, on each KAM variable were assessed using the two factor repeated measures analysis of variance and post-hoc pair-wise comparisons (t-test) with the flat insole condition as a control.

Additionally, percent changes in the KAM, which is frequently used parameter to communicate biomechanical effect of laterally wedged insoles, [12–14, 29] were calculated for the four reference frames. The percent change was defined as the following equation: $\frac{\left(KAM\;in\;the\;laterally\;wedged\;insole\;condition–KAM\;in\;the\;flat\;insole\;condition\right)}{KAM\;in\;the\;flat\;insole\;condition}$

Although most studies used the magnitude of KAM for statistical comparison, [13, 14, 27, 29] Lewinson et al. [30] statistically tested the percent change as well as the absolute value. Therefore we also compared the percent changes among the different frames using the Friedman test and post-hoc pair-wise comparisons (Scheffe). Statistical significance was set at α = 0.05.

## Results

**Fig 1:**
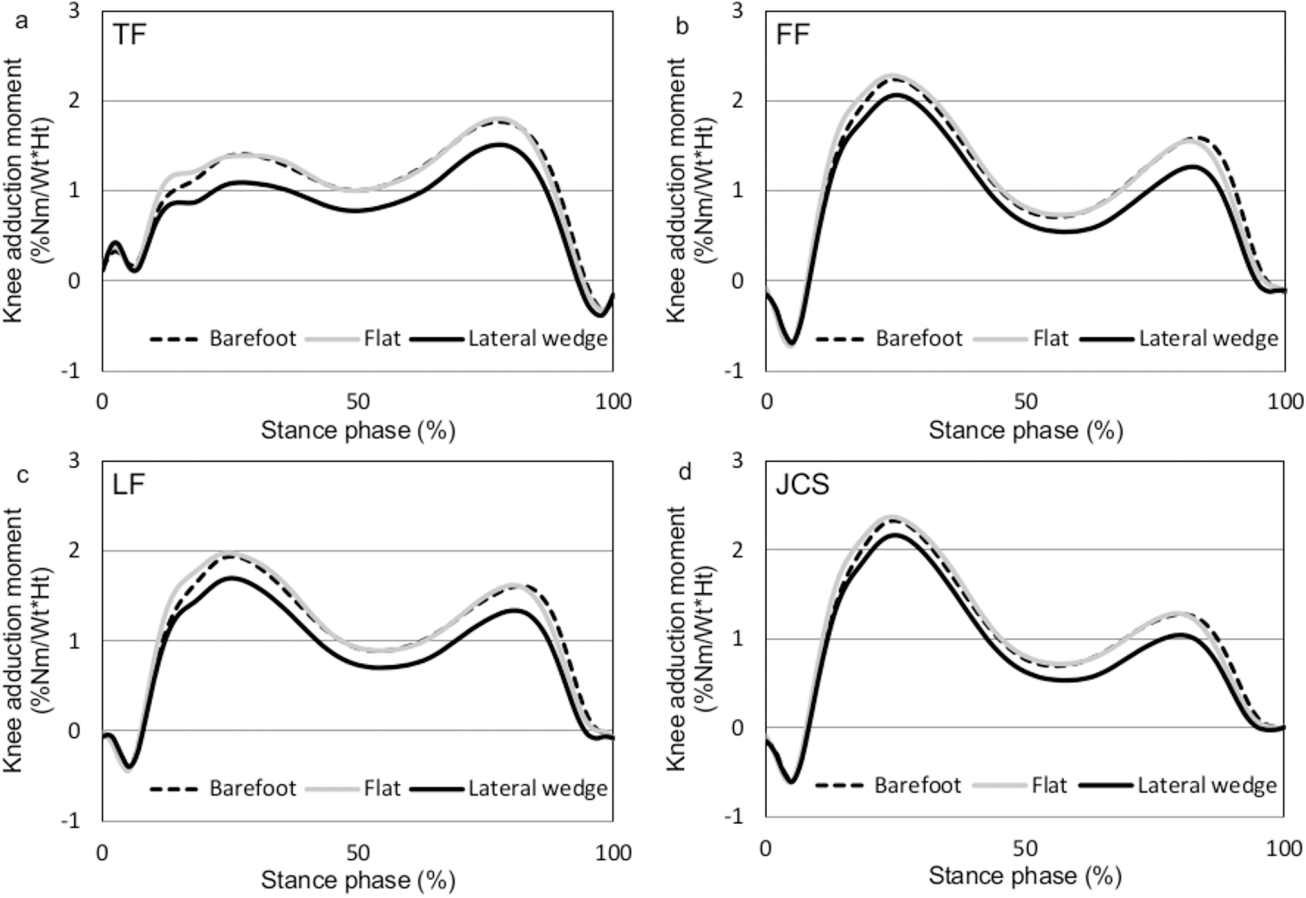
Knee adduction moment curves expressed using the different reference frames. TF, tibial frame; FF, femoral frame; LF, laboratory frame; JCS, Joint Coordinate System.

For all gait conditions, the shapes of the moment waveform were similar when expressed using the different reference frames, and had two peaks in the first and second halves of the gait cycle (Fig 1).

There were significant effects of laterally wedged insole on reference frame on all KAM variables (*P* = 0.005 for the first peak, *P* = 0.004 for the second peak, and *P* < 0.001 for the angular impulse, Table 1). There were also significant effects of reference frame on the KAM first peak and second peak (*P* = 0.005 for the first peak and *P* = 0.004 for the second peak, Table 1). The post-hoc tests showed that the KAM first peak for the TF was significantly smaller than those for the other reference frames, and the first peak for the LF was significantly smaller than those of the FF and JCS. The KAM second peak for the JCS was significantly smaller than those for the other reference frames. The effect of reference frame was not significant for the angular impulse (*P* = 0.84, Table 1). No significant interaction between the gait condition and reference frame was found in any of the KAM variables (*P* = 0.99 for all KAM variables).

**Table 1:** Knee adduction moment parameters expressed using the different reference frames during gait with flat insole and laterally wedged insole.

		TF	FF	LF	JCS	*P value* *
		<0.001				
First peak (%Nm/Wt*Ht)	Flat	1.7±0.7 (1.5–1.9)	2.4±0.7 (2.1–2.7)	2.1±0.7 (1.9–2.4)	2.5±0.8 (2.2–2.7)	0.005
	Lateral wedge	1.4±0.6 (1.2–1.7)	2.2±0.7 (1.9–2.4)	1.9±0.8 (1.6–2.1)	2.3±0.7 (2.0–2.5)	
		0.006				
Second peak (%Nm/Wt*Ht)	Flat	1.9±0.7 (1.6–2.1)	1.6±0.7 (1.3–1.9)	1.7±0.7 (1.4–2.0)	1.4±0.6 (1.1–1.6)	0.004
	Lateral wedge	1.6±0.7 (1.3–1.8)	1.4±0.67 (1.1–1.6)	1.4±0.7 (1.2–1.7)	1.2±0.6 (0.9–1.4)	
		0.84				
Angular impulse (%Nm*Sec/Wt*Ht)	Flat	0.68±0.26 (0.58–0.78)	0.71±0.28 (0.61–0.82)	0.71±0.28 (0.60–0.82)	0.68±0.25 (0.58–0.78)	<0.001
	Lateral wedge	0.54±0.25 (0.45–0.64)	0.50±0.25 (0.50–0.69)	0.58±0.26 (0.48–0.68)	0.57±0.24 (0.48–0.66)	

Values are shown as mean ± standard deviation (95% confident interval).

TF, tibial frame

FF, femoral frame

LF, laboratory frame

JCS, Joint Coordinate System.

*Repeated measure ANOVA.

When the percent change in the KAM was used as a parameter of the efficacy of laterally wedged insole, the mean changes ranged from -10% to -16% for the first peak, from -16% to -18% for the second peak, and from -17% to -21% for the angular impulse when using different reference frames (Table 2). The percent reduction in the first peak for the TF was significantly larger than those for the FF and JCS (*P* < 0.001, Table 2). Significant differences were also found for the second peak and angular impulse (*P* = 0.02 and 0.01, respectively, Table 2), although the post-hoc tests showed no significant differences.

**Table 2:** Percent changes in the knee adduction moment in response to laterally wedged insoles expressed using the different reference frames.

	TF	FF	LF	JCS	*P value* *
First peak (%)	−16.2±13.0 (−11.3 to −21.1)	−10.1±7.7 (−7.2 to −13.0)	−15.0±10.7 (−10.9 to −19.1)	−9.2±7.6 (−6.3 to −21.1)	< 0.001**
Second peak (%)	−15.6±7.6 (−12.8 to −18.5)	−18.0±15.7 (−12.0 to −23.9)	−17.7±12.4 (−13.0 to −22.5)	−17.5±14.8 (−11.9 to −23.1)	0.02
Angular impulse (%)	−21.0±15.6 (−15.1 to −26.9)	−17.9±7.8 (−17.3 to −26.1)	−20.3±10.0 (−16.6 to −24.2)	−17.0±7.6 (−14.1 to −19.9)	0.01

Values are shown as mean ± standard deviation (95% confident interval).

TF, tibial frame

FF, femoral frame

LF, laboratory frame

JCS, Joint Coordinate System.

Horizontal bars indicate statistical differences in post-hoc pair-wise comparisons.

*Friedman test.

**Significant differences between TF and FF, TF and JCS, and LF and JCS in post-hoc pair-wise comparisons.

**Fig 2:**
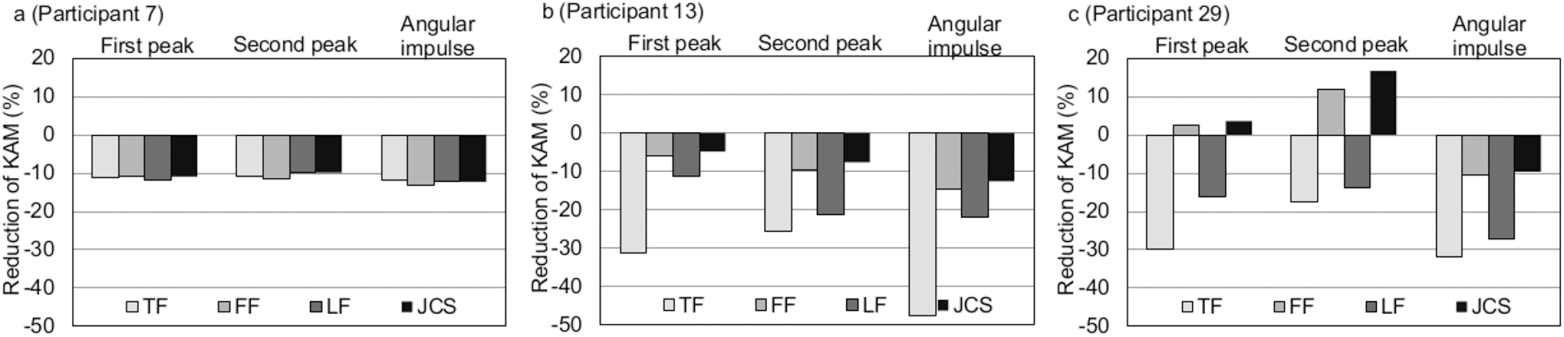
Reduction in the knee adduction moment in response to laterally wedged insoles expressed using the different reference frames. Extreme cases. Participant 7 (a), 13 (b) and 29 (c). TF, tibial frame; FF, femoral frame; LF, laboratory frame; JCS, Joint Coordinate System; KAM, knee adduction moment.

Looking at the data from each participant, the differences in the percent change of the KAM among the reference frames were not consistent across participants. In eleven participants, the differences in the percent change of the KAM first peak between the reference frames were less than 5% (e.g. Fig 2A, participant 7). On the other hand, the differences between the reference frames were more than 10% in 12 participants e.g. Fig 2B, participant 13), indicating that the selection of the reference frame greatly affected the reduction of the KAM in these participants. Five and sixteen participants had more than 10% of differences in the percent change for the KAM second peak and angular impulse, respectively.

The KAM first peak increased in response to the laterally wedged insole in four participants. The KAM second peak and angular impulse increased in one and three participants, respectively. The increases were not consistent across the reference frame. For example, in participant 29, the KAM first peak and second peak increased for the FF and JCS. On the contrary, it decreased for the TF and LF (Fig 2C).

## Discussion

No significant interaction between the gait condition and reference frame was found on any of the KAM variables. This result suggests that the effects of laterally wedged insole on the KAM were similar across the four reference frames in terms of absolute value. The result contradicted our hypothesis that reduction of the KAM would be dependent on the reference frame. Conversely, when using the percent change in the KAM as a parameter, differences among the reference frames were found.

We showed conflicting results between statistical tests for the magnitude of the KAM and those for the percent change of the KAM. This discrepancy occurs because the baseline value of the KAM (KAM in the flat insole condition) as well as the magnitude of change in the KAM can affect the percent change. For example, the magnitudes of change in the KAM first peak were similar among the reference frames (-0.26, -0.26, and -0.30%Nm/Wt*Ht for the TF, FF, and JCS, respectively), while the baseline KAM value was smaller for the TF (1.7%Nm/Wt*Ht) than for the FF and JCS (2.4 and 2.4%Nm/Wt*Ht). As a result, the percent change in the first peak was larger for the TF (-16.2%) than for the FF (-10.2%) and JCS (-9.2%). In a systematic review by Radzimski et al. [12], the average percent reduction in the KAM varied among studies, ranging from 5.6% to 12.6% in healthy participants and 2.1% to 11.9% in patients with knee osteoarthritis. While many factors can affect the percent reduction in the KAM, including the amount of wedge used and knee alignment of the patients, selection of the reference frame might have caused the variability among the studies. Researchers also need to be cautious that the percent change in the KAM may not directly reflect the amount of change.

The percent change in the KAM first peak for the TF was 16%, which was greater by 6% than the percent change for the FF (10%), and greater by 7% than that for the JCS (9%)(Table 2). In this study, the percent changes in the KAM variables were 9% to 21%, and larger than previous reports [12]. Therefore 6 to 7% difference between reference frames in this study might be less comparable to other studies, and clinical significance of the difference among the reference frames needs further research. Several factors can affect the effectiveness of laterally wedged insole, including angle [31] and length [32] of the wedge, and selection of participant (e.g. healthy subjects or patients with knee osteoarthritis) [13]. Furthermore, other factors such as age of participant and gait condition (e.g. gait with or without shoes) may affect the effectiveness. The experimental setting of our study might have been the best case scenario, and thus the percent changes in the KAM were larger than previous studies. Indeed, Kakihana et al. [31] used similar experimental setting to our study: insole with a 6° inclination along full length of the foot, gait without shoes, young and healthy participants. The mean percent change in the KAM of their study was 24%, which was comparable to our results. Nevertheless, we need to recognize that the percent change in the KAM first peak may be larger when expressed using the TF than expressed using the other frames, specifically when we compare the biomechanical effect of laterally wedged insoles between different studies.

Whereas the percent changes in the KAM in response to laterally wedged insoles were consistent across the reference frames in some participants, the changes were variable in other participants; the difference in the percent change between the reference frames within a participant was more than 10% in one third of the participants. In six participants, the effect of the laterally wedged insole was even reversed depending on the reference frame. Although this variability among participants is inherent in human studies, it has been an issue in previous studies [13, 14]. Thus it is worth mentioning these extreme cases. Schache et al. [18] showed a similar case for the KAM reduction by medial thrust gait, and reported that the percent reductions in the first peak ranged from 2.4% to 43.8% depending on the reference frame with which they were expressed. Schache et al. [18] also showed that knee adduction moment was increased by medial thrust gait for the FF, however it was decreased for the LF. Nakajima et al. [27] compared biomechanical effects of different insole designs; however, they might have found different results using different reference frames. Thirteen percent to 18% of people with knee osteoarthritis have an adverse biomechanical response to laterally wedged insoles; increased KAM [13, 29]. However, as was shown in Fig 2C in this study, different reference frames can lead to contrasting interpretations of its effectiveness. Therefore, choice of reference frame is critical when determining the individual effectiveness of treatment intervention, including the use of laterally wedged insoles.

**Fig 3:**
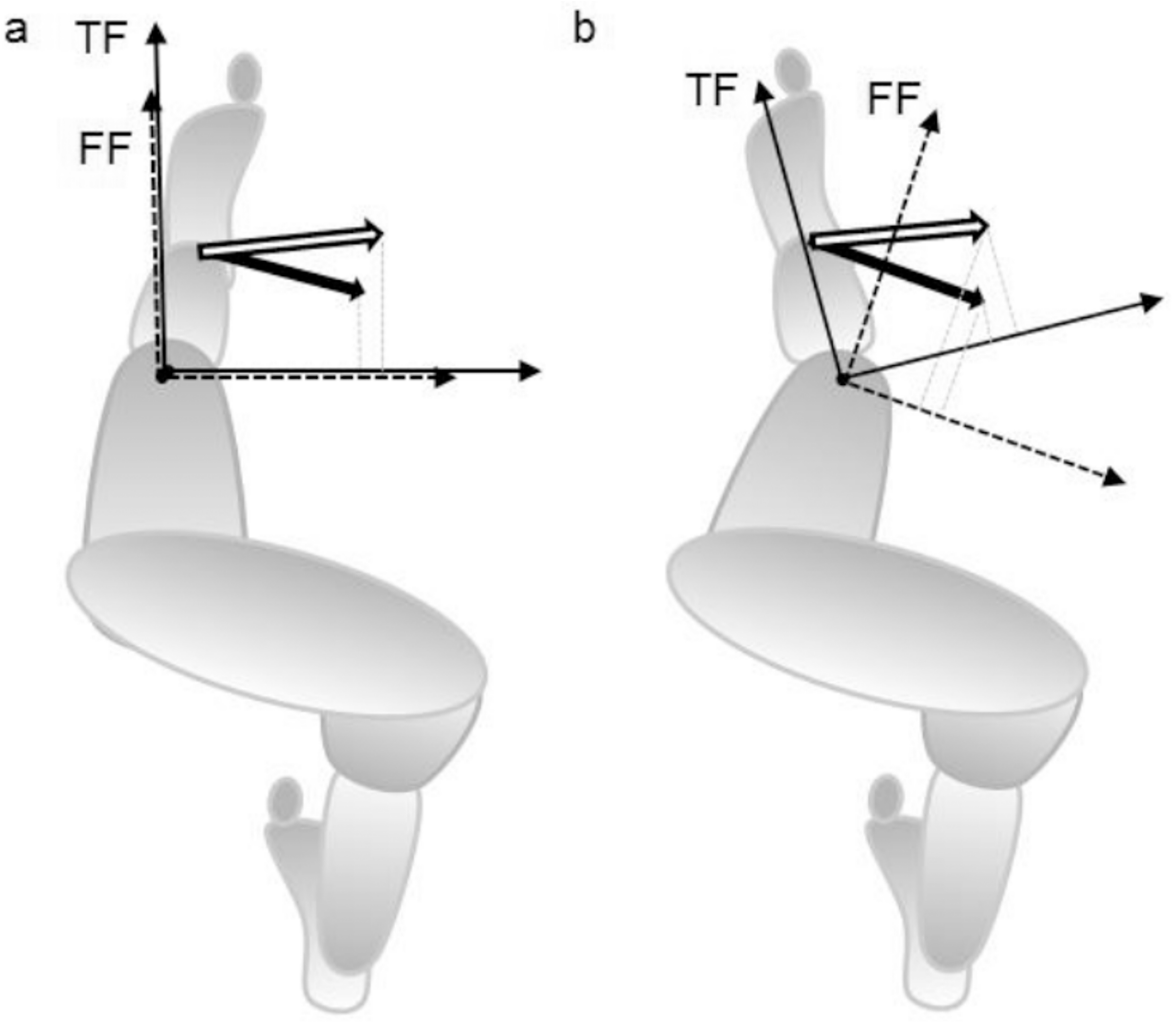
Illustration of reference frames and ground reaction forces in the axial plane. White arrows indicate ground reaction force in the flat insole condition, and black arrows indicate ground reaction force in the laterally wedged insole condition. TF, tibial frame; FF, femoral frame. When the reference frames are aligned parallel in the axial plane (a), the lever arm of the ground reaction force is smaller in the laterally wedged insole condition than in the flat insole condition for both TF and FF. When the reference frames are rotated with each other (e.g. external rotation of the knee)(b), the lever arm in the laterally wedged insole condition is the smaller than in the flat insole condition for TF, while the lever arm in the laterally wedged insole condition is larger than in the flat insole condition for FF.

Rotation of the reference frame in the axial plane can be one of the causes of the variability and reversal in the KAM change. KAM is predominantly caused by the ground reaction force. The lever arm of the ground reaction force along the mediolateral axis of the reference frame affects the calculated KAM [33]. If the reference frames are aligned parallel in the axial plane, as for participant 7 (Fig 3A), the reduction of the KAM in response to laterally wedged insole will be similar regardless of the reference frame used (Fig 2A). However, if reference frames are rotated with each other in the axial plane, as for participant 29 (Fig 3B), the effect of the insole can be reversed (Fig 2C). Further research is necessary to clarify factors affecting the inter-subject variability in the KAM.

There is no consensus regarding which reference frame is the most appropriate to express the KAM. Schache et al. [20] recommended the JCS because it is the most anatomically relevant frame, and it would reflect the muscle function around the knee joint. On the other hand, Winter et al. [34] suggested the use of the LF because the center of mass, as well as the lower limb segments, moves forward in the plane of progression, and therefore the moments that cause those trajectories must be expressed along the plane of progression, or the LF. Since the KAM is regarded as a proxy of the medial compartment contact force, the most appropriate reference frame would be the one with which the KAM best correlates with the medial compartment contact force. Walter et al. [17] examined the correlation between the KAM and the medial contact force using one patient with a force-measuring knee implant. They showed that rotating the tibial frame about the superior axis created large variations in the correlations. However, the ideal frame depends on the target KAM parameter, and there is no single reference frame with which the contact force best correlates all the KAM parameters. We were not able to draw a conclusion about the ideal reference frame, because we did not measure the joint contact force in this study. However, we can state that it is important to specify which reference frame is used to calculate the percent change in the KAM when reporting the biomechanical effect of laterally wedged insoles. We also need to be extremely cautious in comparing between studies in which different reference frames are used. Regarding the choice of KAM parameter, the angular impulse was the most consistent across the reference frames, and may be appropriate to express the KAM.

This study is not without limitations. First, the study was conducted using asymptomatic participants. Although we selected participants with varus knee alignment, it may not be possible to extrapolate the results to patients with medial knee osteoarthritis [12]. Second, the current results are limited within the coordinate systems of the lower limb segments we used. The result might be different if other coordinate systems are used.

## Conclusion

Effects of laterally wedged insole on KAM were similar across the four reference frames. On the other hand, when using the percent change in KAM as a parameter, differences among the reference frames were found. Specifically, the percent change in the KMA first peak was largest when the TF was used, and was smallest when the FF or JCS was used. Researchers need to recognize that the choice of reference frame can significantly influence the interpretation of how laterally wedged insoles affect the KAM when the percent change is used as a parameter.
